# Educational technology for primigravidae: a quasi-experimental study^*^

**DOI:** 10.1590/1980-220X-REEUSP-2022-0040en

**Published:** 2022-06-17

**Authors:** Alexandra do Nascimento Cassiano, Elizabeth Teixeira, Rejane Maria Paiva de Menezes

**Affiliations:** 1 Universidade Federal do Rio Grande do Norte, Departamento de Enfermagem, Natal, RN, Brazil.; 2 Universidade Federal do Pará, Belém, PA, Brazil.

**Keywords:** Obstetric Nursing, Labor Onset, Pregnancy Complications, Educational Technology, Validation Study, Enfermería Obstétrica, Início del Trabajo de Parto, Complicaciones del Embarazo, Tecnología Educacional, Estudio de Validación, Enfermagem Obstétrica, Início do Trabalho de Parto, Complicações na Gravidez, Tecnologia Educacional, Estudos de Validação

## Abstract

**Objective:**

To analyze the influence of an educational technology on the knowledge of primigravidae about the signs of labor and obstetric risk.

**Method:**

A quasi-experimental, before-and-after, non-randomized and non-concurrent study carried out with 90 primigravidae. For data analysis, descriptive statistics was applied,*McNemar*and*Student’s t test*. The project was submitted and approved by the Research Ethics Committee in 2020.

**Results:**

The profile of the pregnant women corresponded to women with an average age of 23 years, brown, living in a common-law marriage, and with an average of 10 years of education. Half of the pregnant women were in the third trimester of pregnancy and were undergoing usual-risk prenatal care. Only 12.22% of the participants reported having access to information on the subject. Data analysis showed a significant difference in the number of correct answers for the questions, before and after viewing the animated video, especially in questions related to signs of labor, bag of waters, and fetal movement test.

**Conclusion:**

Educational technology has a positive influence on primigravidae’s knowledge about signs of labor and obstetric risk.

## RESUMO

**Objetivo:**

Analisar a influência de uma tecnologia educacional sobre o conhecimento de primigestas acerca dos sinais de trabalho de parto e de risco obstétrico.

**Método:**

Estudo quase experimental do tipo antes e depois, não randomizado e não concorrente, realizado com 90 primigestas. Para análise dos dados foi aplicada estatística descritiva, *McNemar* e *t Student*. O projeto foi submetido e aprovado pelo Comitê de Ética em Pesquisa em 2020.

**Resultados:**

O perfil das gestantes correspondeu a mulheres com idade média de 23 anos, pardas, que vivenciam uma união estável e com média de 10 anos de escolaridade. Metade das gestantes estavam no terceiro trimestre gestacional e realizavam o pré-natal de risco habitual. Apenas 12,22% das participantes relataram ter acesso a informações sobre a temática. A análise dos dados evidenciou diferença significativa no número de acertos das questões, antes e depois da visualização do vídeo animado, principalmente nas perguntas relacionadas aos sinais de trabalho de parto, bolsa d’águas e mobilograma.

**Conclusão:**

A tecnologia educacional tem influência positiva sobre o conhecimento das primigestas acerca dos sinais de trabalho de parto e de risco obstétrico.

## RESUMEN

**Objetivo:**

Analizar la influencia de una tecnología educacional sobre el conocimiento de primigestas acerca de las señales de trabajo de parto y de riesgo obstétrico.

**Método:**

Estudio casi experimental del tipo antes y después, no randomizado y no concurrente, realizado con 90 primigestas. Para análisis de los datos fue aplicada estadística descriptiva,*McNemar*y*t* *Student*. El proyecto fue sometido y aprobado por el Comité de Ética en 2020.

**Resultados:**

El perfil de las gestantes correspondió a mujeres con edad media de 23 años, pardas, que están en una unión estable y con media de 10 años de escolaridad. Mitad de las embarazadas estaban en el tercer trimestre gestacional y realizaban el preembarazo de riesgo habitual. Solamente el 12,22% de las participantes relataron tener acceso a informaciones sobre la temática. El análisis de los datos evidenció diferencia significativa en el número de aciertos de las cuestiones, antes y después de la visualización del vídeo animado, principalmente en las preguntas relacionadas a las señales del trabajo de parto, bolsa de aguas y conteo de movimientos fetales.

**Conclusión:**

La tecnología educacional tiene influencia positiva sobre el conocimiento de las primigestas acerca de las señales de parto y de riesgo obstétrico.

## INTRODUCTION

Pregnancy and childbirth are physiological events encompassing biological, psychological, and sociocultural aspects^(1)^. Particularly for primigravidae, the lack of experience living both moments can intensify feelings of doubts, anxieties, and concerns that culminate in unnecessary comings and goings to the health services, due to the difficulty of identifying the exact time to go to the maternity.^(2)^As a result, the woman may be subjected to unnecessary interventions, such as early hospitalization, repetitive digital vaginal examinations, use of oxytocin, amniotomy, longer hospitalization, misdiagnosis of dystocia, and cesarean delivery with no obstetric or neonatal indication^(3,4)^.

Studies on the knowledge of pregnant women about signs of labor and obstetric risk found that women have difficulty differentiating premonitory signs from signs of active labor, in addition to not being able to distinguish complications that are worthy being evaluated during pregnancy^(3,4)^. Moreover, during prenatal care, a significant percentage of women report not having access to information on the subject, especially when considering the North and Northeast regions of the country^(4,5)^.

In this context, Educational Technologies (ET) are considered a light technology^(6)^that, when used by nurses during health education, becomes a learning instrument aiming to qualify their work and favor the educational process^(7)^. Its applicability, specifically in the group of primigravidae, may contribute to the women’s knowledge, self-perception, and autonomy on the subject, therefore contributing to assertive decision-making as to the opportune moment to go to the maternity hospital.

In a review carried out in the literature, the use of different technologies mediating care, such as educational groups, booklets, software, blog, games, manuals, and educational albums^(8)^was observed; however, there are few studies using animated videos as an important educational tool. This resource consists of a complementary informational support that uses texts, sounds, images, and interactive dialogue to provide a multisensory experience and effective learning for those who watch it^(9)^. Additionally, the use of a technology that is easy to disseminate, which can be used both individually, during prenatal consultations, and collectively, in groups of pregnant women, allows easy access to information at the most convenient moment or as many times as necessary.

In view of the possible benefits from ET, it is valid to carry out studies that produce scientific evidence regarding its*in loco*use and its effects on the population^(10)^.

Therefore, the objective of this study was to analyze the influence of an educational technology on the knowledge of primigravidae about the signs of labor and obstetric risk before and after viewing the animated video. Thus, the influence animated videos exert on pregnant women’s knowledge about the subject is the object of this study.

## METHOD

### Design of Study

This is a quasi-experimental, before-and-after, non- randomized, non-concurrent study. This type of study can also be called a historical study, as it makes a comparison in time, where subjects are evaluated before and after participating in an action^(11)^. Thus, the primigravidae underwent an intervention, represented in this study by the animated video on signs of labor and obstetric risk. The video script was elaborated by the researcher based on a literature review, and, once finished, was validated by expert judges, obtaining a CVI value of 0.97 and*kappa*of 0.55. The media development was implemented thanks to the joint work of the researcher and graphic design professionals and the entire production process took place through the stages of script elaboration, storyboard, pre-production, animation, and finalization. At the end, the final product was a full HD animated video with translation into the Brazilian Sign Language (LIBRAS) and duration of about 5 minutes, entitled: “To a first-time mom: when to go to the maternity hospital?”.

To analyze the influence of the video on the knowledge of the participants, the pre-test and the post-test were carried out before and after access to it, to quantify the women’s number of correct answers.

### Population

The study population consisted of primigravidae residing in the city of Natal/RN and who underwent prenatal care in Primary Health Care (PHC).

### Local

The research location corresponded to the Primary Family Health Units (*UBSF*) in the city of Natal, Rio Grande do Norte (RN), Brazil, linked to the Family Health Strategy (FHS).

The definition was based on Cluster Sampling (CS), with selection through Simple Random Sampling (SRS). Thus, 20*UBSF*were randomly selected considering a total of 40 units. The establishment of the number of units was based on a study carried out with pregnant women to validate similar technology^(12)^, and the draw was performed by the researcher using the Microsoft Excel.

### Selection Criteria

The study participants were pregnant women undergoing prenatal care at the*UBSF*, at any period of pregnancy, aged over 15 years and who were primigravidae. Pregnant women with comorbidities and who underwent High Risk Prenatal Care (*PNAR*) could also be included, in addition to secundigravidae with a history of abortion, but who have not yet experienced their first childbirth. Women pregnant with twins and multiparous women were excluded.

### Sample Definition

The sample size calculation was established using the G Power software, version 3.1.9.2. Considering a Cohen effect size of 0.30, test power of 0.80 and a significance level of 5%, a sample of 90 primigravidae from different gestational trimesters was obtained^(13)^.

### Data Collection

Data collection was preceded by the request for the Letter of Consent (CA) to the co-participating institution and project approval by the Research Ethics Committee (*CEP*). After establishing the sample number, the recruitment of pregnant women at the *UBSF* was defined for convenience, on the days and times scheduled for prenatal consultations at the units, during the months of July to October 2021. In each unit, data were collected from four to six pregnant women, to reach the pre-established sample number (90 participants).

The access to the animated video took place within the scope of the health service, preferably in a private place and previously agreed with the managers of each unit, with use of a Samsung tablet, provided by the researcher. Due to the COVID-19 pandemic, preventive measures were adopted, such as the use of masks and the distance of 2 meters during the interviews. The pen used to sign the Free and Informed Consent Form (FICF) was provided by the researcher, preceded by the use of 70% alcohol by the pregnant women, and the handling of the tablet used to view the video was limited to the person responsible for the research.

For the collection, an instrument validated in a previous study was used, consisting of the following elements: identification, health conditions, past obstetric history, current obstetric history, and questions related to knowledge about signs of labor and obstetric risk^(4)^. The result regarding the primigravidae’s knowledge on the subject was represented by the average number of correct answers, which ranged from 1 to 10. At the end of the instrument, there is also an assessment of the level of importance that the animated video represented for primigravidae, using a numerical scale.

Participants were invited to answer the questionnaire based on their knowledge and previous experiences, and then watched the video. Finally, they answered the questionnaire again, and they could change the answer. This way, it was possible to observer whether there was a change in the number of correct answers before and after the intervention.

### Data Analysis and Treatment

The database was built in the Microsoft Office Excel 2020 Statistical tests were applied using the free software *R* 4.2.0

The analysis of qualitative variables was performed using descriptive statistics with absolute and relative frequency distributions. While in the quantitative variables, measures of trend and data dispersion were analyzed, such as minimum, maximum, mean, and standard deviation. To compare the number of the pregnant women’s correct answers, before and after the intervention, statistical tests of McNemar and t student were applied. For all statistical tests applied, the significance level was 5%.

### Ethical Aspects

The research was submitted to the CEP of the Universidade Federal do Rio Grande do Norte (*UFRN*) and followed the recommendations of Resolution 466/2012 of the National Health Council. It was approved in 2020 with Opinion No. 4.396.786. All research subjects signed the FICF.

The risks involved with the participation of pregnant women were the possibility of exposing data related to health and obstetric history. In addition, access to information about possible changes and complications during pregnancy could also generate discomfort. To minimize such risks, the participants were informed about the research procedures, and they were ensured the right to withdraw their consent at any time.

## RESULTS

The profile of the primigravidae who participated in the study corresponded to women with a mean age of 23 years (SD = 5.93), brown (64.45%) and who are in a common law marriage or marriage (74.44%). The participants’ years of education averaged 10 years, which corresponds to elementary school, and the family income of more than half of the pregnant women (54.45%) was 1 minimum wage. Of the pregnant women, 62.22% had no paid occupation, were housewives. The Table 1 below presents the characterization of the participants’ sociodemographic variables.

**Table 1.** Characterization of the primigravidae’s sociodemographic variables – Natal, RN, Brazil, 2022.

Variables		N (%)
**Age**	up to 18 years	21 (23.33)
	From 19 years	69 (76.67)
**Race**	Brown	58 (64.45)
	White	20 (22.22)
	Black	11 (12.22)
	Yellow	1 (1.11)
**Marital status**	Married/Common law marriage	67 (74.44)
	Single	23 (25.56)
**Family income**	Below 1 minimum salary	18 (20.00)
	1 minimum salary	49 (54.45)
	2 minimum salaries	19 (21.11)
	3 minimum salaries	4 (4.44)
**Occupation**	Housewife	56 (62.22)
	Self-employed	5 (5.57)
	Receptionist	5 (5.57)
	General services assistant	3 (3.33)
	Teacher	3 (3.33)
	Housekeeper	2 (2.22)
	Sales person	2 (2.22)
	Cashier	2 (2.22)
	Kitchen assistant	2 (2.22)
	Other	10 (11.10)
**Total**		90 (100)

Data collected by the author.

Of the pregnant women interviewed, 23.33% reported a family history of pathologies such as Systemic Arterial Hypertension (SAH) (82.61%) and Diabetes mellitus (71.01%). Regarding personal history, one (1.11%) pregnant woman reported using alcohol, three (3.33%) were smokers, and two (2.22%) used drugs. As provided for the ministerial protocols, they used ferrous sulfate (83.78%) and folic acid (68.92%) during pregnancy. Regarding the obstetric history, 74.44% were primigravidae and 25.56% had already experienced abortion, but not childbirth. It should be noted that 50% of the interviews were in the course of the third trimester of pregnancy (27 to 41 weeks), while 34.44% were in the second trimester (14 to 26 weeks) and 15.56% were in the first trimester (1 to 13 weeks). Although 85.56% of pregnant women were on usual-risk prenatal care, a percentage of pregnant women performed high-risk prenatal care (*PNAR*) for Gestational Diabetes Mellitus (GDM) (33.33%) and for Gestational Hypertensive Syndrome (GHS) ) (25%).

During prenatal care at PHC, only 4.44% of pregnant women participated in some group educational activity and 12.22% reported having access to information about signs of labor and obstetric risk. When such guidelines were received, they were mostly provided by the physician (7.78%), followed by the nurse (2.22%).

According to Table 2, data analysis using McNemar test showed a statistically significant difference (p < 0.0005) between before and after the animated video in questions 1 (premonitory signs of labor), 2 (mucous plug characteristics), 3 (bag of waters rupture), 4 (amniotic fluid characteristics), 5 (what to do when the bag of waters breaks), and 6 (number of contractions that indicate the beginning of labor).

**Table 2.** Assessment of primigravidae’s knowledge before and after viewing the animated video (paired comparison) – Natal, RN, Brazil, 2022.

Question	Before	After N (%)		Total N (%)	p-value^(1)^
		Got wrong answers	Got right answers		
**Q1**	**Got wrong answers**	14 (27.45)	37 (72.55)	51 (100.00)	<0.001
	**Got right answers**	1 (2.56)	38 (97.44)	39 (100.00)	
**Q2**	**Got wrong answers**	9 (29.03)	22 (70.97)	31 (100.00)	<0.001
	**Got right answers**	—	59 (100.00)	59 (100.00)	
**Q3**	**Got wrong answers**	1 (10.00)	9 (90.00)	10 (100.00)	0.004
	**Got right answers**	—	80 (100.00)	80 (100.00)	
**Q4**	**Got wrong answers**	2 (12.50)	14 (87.50)	16 (100.00)	0.004
	**Got right answers**	2 (2.70)	72 (97.30)	74 (100.00)	
**Q5**	**Got wrong answers**	9 (36.00)	16 (64.00)	25 (100.00)	<0.001
	**Got right answers**	—	65 (100.00)	65 (100.00)	
**Q6**	**Got wrong answers**	4 (5.63)	67 (94.37)	71 (100.00)	<0.001
	**Got right answers**	—	19 (100.00)	19 (100.00)	
**Q7**	**Got wrong answers**	—	49 (100.00)	49 (100.00)	—
	**Got right answers**	—	41 (100.00)	41 (100.00)	
**Q8**	**Got wrong answers**	—	1 (100.00)	1 (100.00)	—
	**Got right answers**	—	89 (100.00)	89 (100.00)	
**Q9**	**Got wrong answers**	—	2 (100.00)	2 (100.00)	—
	**Got right answers**	—	88 (100.00)	88 (100.00)	
**Q10**	**Got wrong answers**	2 (28.57)	5 (71.43)	7 (100.00)	0.063
	**Got right answers**	—	83 (100.00)	83 (100.00)	
**Total**			90 (100.00)	90 (100.00)	

Data collected by the author. ^(1)^McNemar.

According to the data, the pregnant women who got wrong answers in the first evaluation were more correct in the second evaluation. This means that after viewing the animated video, their performance in solving the questions was better, which suggests a positive influence on the level of knowledge, especially on the signs of labor. The detailed interpretation of the data distributed in the table below suggests that, for example, in question 1, 27.45% of the pregnant women who were wrong in the first assessment remained wrong in the second assessment even after viewing the video. On the other hand, 72.55% of the pregnant women who got it wrong before got it right after the intervention. The same reasoning applies to the other questions.

Finally, in Table 3, through the Student’s t test, it can be observed that there was a statistically significant difference (p < 0.001) between before and after viewing the animated video in the number of correct answers for the questions. Thus, the data suggest that the primigravidae had a greater total number of correct answers to the questions after the intervention.

**Table 3.** Assessment of primigravidae’s knowledge before and after viewing the animated video (Number of correct questions) – Natal, RN, Brazil, 2022.

Time	Minimum	Maximum	Mean	SD	p-value^(2)^
**Before**	3.00	10.00	7.0	7.08	<0.001
**After**	5.00	10.00	10.00	0.88	

Data collected by the author. ^(2)^Paired Student’s t test.

In addition, in the evaluation of primigravidae regarding the importance of the video presented, on a scale from 0 to 10, the average grade attributed was 9.92 (SD = 0.34), with a minimum of 7 and a maximum of 10.

## DISCUSSION

The results presented indicate that the ET, in animated video format, had a positive influence on the knowledge of primigravidae about the signs of labor and obstetric risk. This is because the pregnant women’s average of correct answers was higher after viewing the video, with a statistically significant difference when compared to the number of correct answers before accessing it.

With the study, a profile of primigravidae could be observed, showing women with an average age of 23 years, of brown color, living in a common law marriage or in a marriage, with completed elementary school, housewives and with a family income of 1 minimum wage. Data from the Department of Informatics of the Brazilian Health System (DATASUS) corroborate the characterization found, since, in 2019, of the pregnant women with live births in the city of Natal, 22.42% were aged between 20 and 24 years; 50.07% studied from 8 to 11 years (elementary school); 53 (30%) were in a common law marriage or marriage; and 63 (56%) declared themselves to be brown^(14)^.

At the national level, the Birth in Brazil (*Nascer no Brasil*) survey, carried out with more than 23,000 individuals, also found a similar profile of pregnant women. The participants had an average age of 25 years, were brown, completed elementary school, and lived with a partner^(5)^. Updated data from the same study confirm the results found, with the same sociodemographic characteristics^(15)^. It is considered that the sample of this research has a profile consistent with the national population of Natal. Thus, the positive results with this group suggest the suitability of the technology for use with primigravidae from different realities.

A fact that drew attention concerns the small percentage of women who reported having received guidance regarding the signs of labor and obstetric risk. A study on educational activities with pregnant women during prenatal care found that there is still a lack of guidance in this service, demonstrating a failure of health services to provide information necessary for the promotion of maternal and neonatal health^(16)^. When carried out, there is a tendency in the most prevalent type of guidelines, such as the adoption of healthy eating habits, non-consumption of drinks and drugs and, mainly, on breastfeeding^(17)^.

The relevance of the aforementioned guidelines is acknowledged. However, it is clear that the approach to signs of labor and obstetric risk are commonly neglected. Another work carried out with pregnant women, specifically on the subject, identified a significant association between the performance of the participants in solving issues related to the subject and the guidelines received during prenatal care^(4)^. These results confirm the relevance of the ET produced as an innovative technology to be included in prenatal care.

The Ministry of Health (MH), through the technical manuals for low- and high-risk prenatal care, recommends that, from the first consultation, health education guidelines should be carried out not only in the first consultation, but throughout the entire pregnancy^(18,19)^. Considering the above, it is assumed that health professionals are responsible for informing pregnant women about the subject, so that such knowledge is disseminated during prenatal care^(4)^. Despite this, more than half of the pregnant women were in the third trimester without having received information about signs of labor and obstetric risk. Of the total sample, 87.78% of the participants reported not having access to this type of information, a value higher than another survey that identified a percentage of 61% of pregnant women who did not receive such guidance^(4)^.

Faced with possible difficulties encountered during prenatal follow-up, the professionals who make up the health team, including physicians and nurses, shall be open to new strategies that accompany social changes and optimize the care provided^(18.19)^. In this context, ETs are potential instruments to lead to the pregnant women’s understanding and to assist in addressing issues such as: attention to the elimination of vaginal fluids, signs of labor, fetal movement, presence of contractions, vaginal bleeding, headache, visual disorders, epigastric pain, and other^(18,19)^.

The inferential analysis of the study showed that there was a significant difference between the before and after the animated video, mainly in the questions related to premonitory signs of labor, characteristics of the mucous plug and amniotic fluid, rupture of the bag of waters, number of contractions that indicate the beginning of labor and the fetal movement test. Therefore, the validated technology had a positive influence on the pregnant women’s knowledge on the subject.

Research carried out with pregnant women has identified that women are still unaware of obstetric warning signs and have difficulty differentiating premonitory signs and signs of the active phase of labor, confusing the exact moment to go to the maternity hospital.^(3,4)^ A study carried out, in which the same instrument of this research was used, identified an average of 4.9 hits (SD = 2.0) with a higher percentage of errors in the following questions: signs that precede labor (68%), rupture of the bag of waters (63%), characteristics of the amniotic fluid (65%), number of contractions indicating the beginning of labor (58%) and perception of changes in fetal movement (68%). On the other hand, 90% recognized vaginal bleeding and 87% were able to distinguish symptoms from complications that need to be evaluated during pregnancy^(4)^.

Confirming the data of the thesis presented, there is a pattern in the most wrong questions, which refer to preliminary signs, identification, and behavior in relation to the ruptured bag of waters, changes in fetal movement, and the pattern of uterine contractions that indicate active labor. Similarly, obstetric warning signs, except for changes in fetal movement, apparently are the knowledge that is most consolidated by pregnant women.

During pregnancy and childbirth, many women may be subject to a condition of vulnerability, related, for example, to the lack of information on the differentiation between latent and active phases of labor. This reality is even more fragile in primigravidae, due to their lack of experience regarding childbirth, which generates feelings of doubts, anxiety, fears, and concerns^(16)^. In view of this, education for pregnant women aims to reduce such negative repercussions through strategies that enable the individuals to self-knowledge and control of decisions related to their lives^(16)^.

Therefore, the video developed contributes to the self- perception of signs of labor and obstetric risk, which will determine the timely search for the maternity hospital. This assertive decision will avoid unnecessary comings and goings, and reduce premature admissions, shortening hospital stays and unnecessary interventions during labor^(4)^. It is expected that the experience with technology favors the ability to self-manage and consolidate their conscious participation in decisions that concern them^(20,21)^. Once empowered, the experience of pregnancy takes place autonomously, with independence, self-sufficiency, and freedom^(10)^.

Although the average of correct answers (7.0 SD = 7.8) in the pre-test was higher than that found in another study^(4)^, it is worth noting that, in the post-test, after viewing the video, the average of correct answers changed to 10.0. It is not by chance that technology was considered important by the participants with an average score of 9.92 (SD = 0.34).

In recent years, the internet and the media have become an important source of information with popular access to digital platforms such as Instagram®, Facebook® and WhatsApp®. They became vehicles widely used by population groups of different social classes and levels of education^(22)^. Above all, it is pertinent to invest in public health promotion policies that are intermediated by the exploitation of valued resources in the context of users and the community, such as the animated video^(9,22)^.

Videos are considered the main basis for the dissemination of audiovisual language, as they make visible and concrete, through the senses, the message that is intended to be transmitted. They are widely used in all areas for recording and documenting content with different purposes, as they have characteristics that make it an effective form of communication with the ease of allowing the review of the media product and guaranteeing access when convenient^(23)^.

From this perspective, it is believed that the video is an interesting resource that, when incorporated in a complementary way to the interventions and guidelines already carried out in prenatal care, can contribute to the health care of primigravidae.

The innovative character that this ET brings consists of the manifestation of levels of consciousness, to the detriment of the simple transfer of information. It is made by professional *praxis*, understood as a conscious, oriented, scientific activity with well- defined objectives that transform man himself and those who have access. Thus, technology becomes an intermediary of caring and educating, whose processes are established in an inseparable interrelationship in the search for the individuals’ empowerment, autonomy, and well-being^(24)^. It is a pedagogical methodology that aims to strengthen care and learning of pregnant women.^(24)^, in addition to systematizing assistance with the standardization of the guidelines offered^(9)^.

It is well known, according to the law on professional practice and the public policies that govern PHC, low-risk prenatal care can be monitored by the nurse, who is responsible for carrying out, among other activities, health education actions for pregnant women during the nursing consultation^(3)^. Moreover, this professional is expected to be a reference for health promotion activities, given the proximity of care and education.

The results of the thesis showed a higher percentage of pregnant women who were advised about the signs of labor and obstetric risk by the physician. A study on the association between the best guidance during prenatal care and the professional who assists him found that performing prenatal care together with the doctor and the nurse increased the chances of adequate guidance, when compared to monitoring by only one professional^(25)^. These findings lead to two important reflections. The first concerns the nurses’ need to empower themselves with technologies that help them in the work process, and strengthen them as a member of the team; and the second reinforces the importance of all professionals who assist pregnant women.

Finally, the relevance of technologies and professionals for adequate prenatal care is reaffirmed, in the search for improvements in maternal and child health care and to promote women’s leading role.

## CONCLUSION

The results suggest that ET had a positive influence on primigravidae’s knowledge about signs of labor and obstetric risk. This is because a statistical difference was evidenced between the before and after the animated video, both in the total performance of the resolution of the questions, and in specific questions. This means that after viewing the animated video, their performance in solving the questions was better, which suggests a positive influence on the level of knowledge, especially on the signs of labor. The video was evaluated by the pregnant women regarding its importance, with an average score of 9.92.

As limitations, there is the failure to carry out a previous field study, whose objective would be to recognize with pregnant women which contents and technologies are most appropriate to their needs. Despite this, it should be noted that the choice for the theme, as well as the animated video as an educational technology, was based on a literature review. Another limitation is the fact that, in the quasi-experiment stage, although the selection of the UBSF was carried out by random drawing, the recruitment of participants in each unit was made for convenience on the days of prenatal consultation and according to the researcher’s collection schedule.

Finally, it is believed that the animated video will facilitate pregnant women’s access to information regarding signs of labor and obstetric risk during prenatal consultations and as many times as they deem necessary, since this technology can be accessed on the smartphone or digital media. Therefore, the material can be an instrument that will contribute to the knowledge of primigravidae on the subject and, consequently, will allow autonomy and security in the decision to go to maternity hospital at the opportune and necessary moment. In addition, it will also contribute to the work of primary care professionals and maternity hospitals, in their work with this clientele, by promoting maternal and neonatal health through health education.

## ASSOCIATE EDITOR

Maria Luiza Gonzalez Riesco
